# The effect of community water fluoridation cessation on children's dental health: a national experience

**DOI:** 10.1186/s13584-022-00514-z

**Published:** 2022-01-28

**Authors:** Guy Tobias, Findler Mordechai, Chackartchi Tali, Bernstein Yaron, Greenberg Parizer Beatrice, Mann Jonathan, Sgan-Cohen Harold

**Affiliations:** 1 Department of Community Dentistry, Hebrew University-Hadassah School of Dental Medicine, Jerusalem, Israel; 2 Dental Research Unit – Maccabi-Dent, Maccabi Healthcare Fund, Tel Aviv, Israel; 3 Department of Periodontology, Hebrew University-Hadassah School of Dental Medicine, Jerusalem, Israel

**Keywords:** Community water fluoridation, Public Health Dentistry

## Abstract

**Background:**

Community water fluoride (CWF) is the proven cornerstone of primary dental health care promotion. In 2002 CWF was made mandatory at a national level in Israel, however a new government revoked these regulations in August 2014. "Maccabi" is the second largest national health care provider with 2.3 million members, "Maccabi-Dent", its dental branch, has 53 clinics, employing 1100 dentists. The aim of this study was to evaluate the cumulative effect on treatment rates 6 years after CWF was terminated in Israel, based on the number of dental treatments provided to children aged 3–12 years in “Maccabi-Dent” clinics.

**Methods:**

For this retrospective study, computerized dental treatment codes were collected. The “rate of treatment” was calculated by dividing the number of restorative treatments or extractions, by the number of individuals receiving treatment. The population size and the age group visiting the specific clinic were also considered.

**Results:**

The independent variables were fluoride concentration in drinking water, age and socioeconomic position (SEP). There was a significant increase in restorative dental treatments after 2014, (R^2^ = 0.0402), with approximately twice the number of treatments required in the absence of CWF. Age had a significant association (β =  − 0.389, *p* < 0.001) as did SEP (β = 0.086, *p* = 0.019).

**Conclusion:**

After CWF cessation in Israel, rates of dental treatments significantly increased.

**Practical implication:**

By examining accepted notions with up-to-date information, new confirmatory evidence helps decision makers understand the importance of adding fluoride to drinking water.

## Background

“Fluoridated water contains fluoride at a level that is effective for preventing cavities." [1] In Israel, the recommended optimal fluoride concentration ranges from 0.7 to 1.2 parts per million (ppm) depending on the local temperature and water intake [2]. Other countries have a fluoride concentration between 0.5 and 1.5 ppm [3, 4]. The U.S department of Health and Human Services recommends 0.7 parts per million [5, 6]. The 2011 WHO Guidelines for Drinking-water Quality suggests concentrations between 0.5 and 1 ppm, with an upper safe level of 1.5 ppm [7].

Dental caries remains the most common chronic childhood disease in the US and many other countries. Fluoride has been proven to be a very effective primary care measure in caries prevention [8]. In 2007 over 300 million people in 39 countries lived in optimally fluoridated areas (more than 5% of the world population), [9]. Some of these countries include Brazil, Australia, Canada, Spain, Argentina, South Korea, and New Zealand.

In the US, 74.6% of the population (211 million people) have access to fluoridated water [10].

While removing water fluoridation affects the entire population, those with lower incomes, poorer oral health and significant barriers to dental care, suffer more than those with a higher socio-economic position (SEP) [11]. Israeli studies [12, 13] found an opposite correlation between SEP and caries: the lower the SEP, the higher the level of dental needs.

Another Israeli study by Klivitsky et al., 2015 [14] reported a clear association between adequacy of water fluoridation and hospitalization due to dental infections in children and adolescents. This effect was more prominent in populations with lower SEP.

Fluoridation began at the municipal level in 1981, legislation for national fluoridation passed in 1998, yet, similarly to other countries, there were campaigns against fluoridation in Israel [15]. The Ministry of Health convinced the decision makers that water fluoridation was safe, effective, relatively cheap and data collected showed that the dental caries experience in Israeli children decreased. Fluoridation became mandatory in Israel in 2002 and 75% of the population had fluoridated water with a plan to increase the level to 85% [16].

Following many years of failed attempts to implement "voluntary" fluoridation by local authorities, Israel implemented mandatory national fluoridation legislation in 2002. Public controversy and persistent opposition led to challenges, even after mandatory fluoridation had been legislated by the Israeli parliament. The debate was brought to the High Court which reconfirmed the Ministry of Health's responsibility for the health of the public and that no evidence presented, indicated that fluoridation was not a safe and reasonable measure [17].

An editorial by Lennon et al. [18] published in 2013 in the journal of Community Dental Health strongly supported water fluoridation in Israel. However, when a new government was established in 2014, the regulations were revoked, and water fluoridation was discontinued. Despite a decision made by the Health Ministry of Health in June 2015 and approved by the parliament budgetary issues stalled the fluoridation process. Currently, the drinking water in Israel is not fluoridated. Furthermore, increased reliance on desalination for drinking water means that there are even lower fluoride levels [19].

According to the American Dental Association, fluoridation was meant to return in 2016 [20]. The new government and a new Ministers strongly supported fluoridation, and dental public health professionals joined the fight against the ongoing delays.

In this article we evaluate the outcomes of 6 years without water fluoridation in Israel.

For clarity, a brief review of the Israeli medical and dental care system is presented. All citizens are provided medical care by four medical health funds (HMO). The largest, Clalit Healthcare Services, has almost 5 million members, Maccabi Healthcare services is the second largest with 2.3 million members. Each HMO has independent dental care facilities, that are also government funded. Approximately 50% of Dental care is provided by the HMO's and the rest by private dental services. "Maccabi-dent" has 53 dental clinics and employs 1100 dentists [21]. The 53 clinics are spread throughout the country and with different water fluoridation levels and SEP. The wide geographical distribution of the clinics strengthens the assumption that the children seen in Maccabi-dent are representative of the child population of Israel. Israeli residents choose their own HMO [22]. All the health funds provide free dental care for individuals up to 18 years of age [23].

## Methods

The present study was approved by the Institutional Review Board (IRB) MHS-0157-20 The Helsinki committee of Maccabi Healthcare Services.

Dental treatment patterns between 2014 and 2019 were examined retrospectively. Clinics were divided into geographical areas where community water fluoridation (CWF) was discontinued (intervention group) and areas that never had optimally fluoridated drinking water (the control group). The data was retrieved from the computer system of the 53 "Maccabi-Dent" clinics in Israel. For children, "Maccabi-Dent" dentists are remunerated according to the Fee for Service method, thus each procedure has a unique treatment code, and all codes are recorded for all patients [24].

After collection, the codes were grouped into restorative treatments (e.g., amalgam or tooth-colored fillings, pulpotomy, pulpectomy, crowns etc.) and extractions. each tooth could potentially get only one code regardless of the restorative treatments made on that same tooth. The “rate of treatment” was calculated by dividing the number of restorative treatments or extractions, by the number of individuals receiving treatment.

The independent variables were CWF levels (obtained from the 2011 Ministry of Health Water Engineer's Report) [25], age and socioeconomic status (obtained from the data supplied by “Maccabi-Dent”). As in other published Israeli studies [26], we divided water fluoride levels into three subgroups: No fluoride 0–0.5 ppm; partially fluoridated 0.51–0.69 ppm; optimally fluoridated 0.7–1.2 ppm. The geographic area and fluoride water concentration was determined for each clinic. No fluoride and partially fluoridated were operationally combined as "no fluoride".

Inclusion criteria: 1. Age (3–12 years); 2. Available records from 2014 to 2019. Exclusion criteria: 1. Region where fluoride concentration was unknown; 2. Erroneous or illogical dental records.

### Statistical methods

Data were analyzed with IBM SPSS statistics software version 27.0. (SPSS Inc. Headquarters, 233 S. Wacker Drive, 11th floor Chicago, Illinois 60606, USA).

Statistical significance levels were set at 0.05.

Baseline characteristics are presented as means and standard deviations for continuous variables and as frequencies and percentages for categorical variables. Differences between number of treatments and fluoridation levels were evaluated using Spearman correlation.

Linear regression tests were applied on predictive variables {Age, Socioeconomic Position (SEP) and fluoride levels}.

## Results

### Descriptive data

The total number of routine examinations was 847,548, with an age range of 3–12 years and a mean of 7.5 ± 2.87.

SEP ranged ordinally from 1 to 9 in accordance with the Israel Bureau of Statistics. The mean was 5.69 ± 2.07.

The proportion of children who resided in optimally fluoridated regions was 63.3%; 16.3% resided in partially fluoridated regions; and 20.4% in non-fluoridated regions.

Table 1 presents the mean levels of treatment (restorative, extractions and total) for each of the years 2014–2019 (note that fluoridation ceased in 2015).

**Table 1:** Distribution of recorded treatments by year

	Treatment	Mean	SD	Min	Max
2014	Total	0.64	1.70	0.01	3.65
	Extractions	0.40	2.18	0.00	4.60
	Restorative	0.88	1.29	0.01	2.70
2015	Total	0.56	0.36	0.02	5.13
	Extractions	0.29	0.37	0.00	5.14
	Restorative	0.83	0.49	0.04	6.25
2016	Total	0.61	0.29	0.01	1.74
	Extractions	0.24	0.16	0.00	0.79
	Restorative	0.99	0.55	0.01	3.24
2017	Total	0.65	0.34	0.02	2.41
	Extractions	0.24	0.16	0.00	0.78
	Restorative	1.06	0.64	0.02	4.42
2018	Total	0.65	0.34	0.01	2.23
	Extractions	0.24	0.16	0.00	0.84
	Restorative	1.07	0.64	0.01	4.07
2019	Total	0.66	0.38	0.01	2.56
	Extractions	0.25	0.16	0.00	0.98
	Restorative	1.08	0.71	0.01	4.71
All years except 2014	Total	0.62	0.30	0.02	1.74

**Fig. 1:**
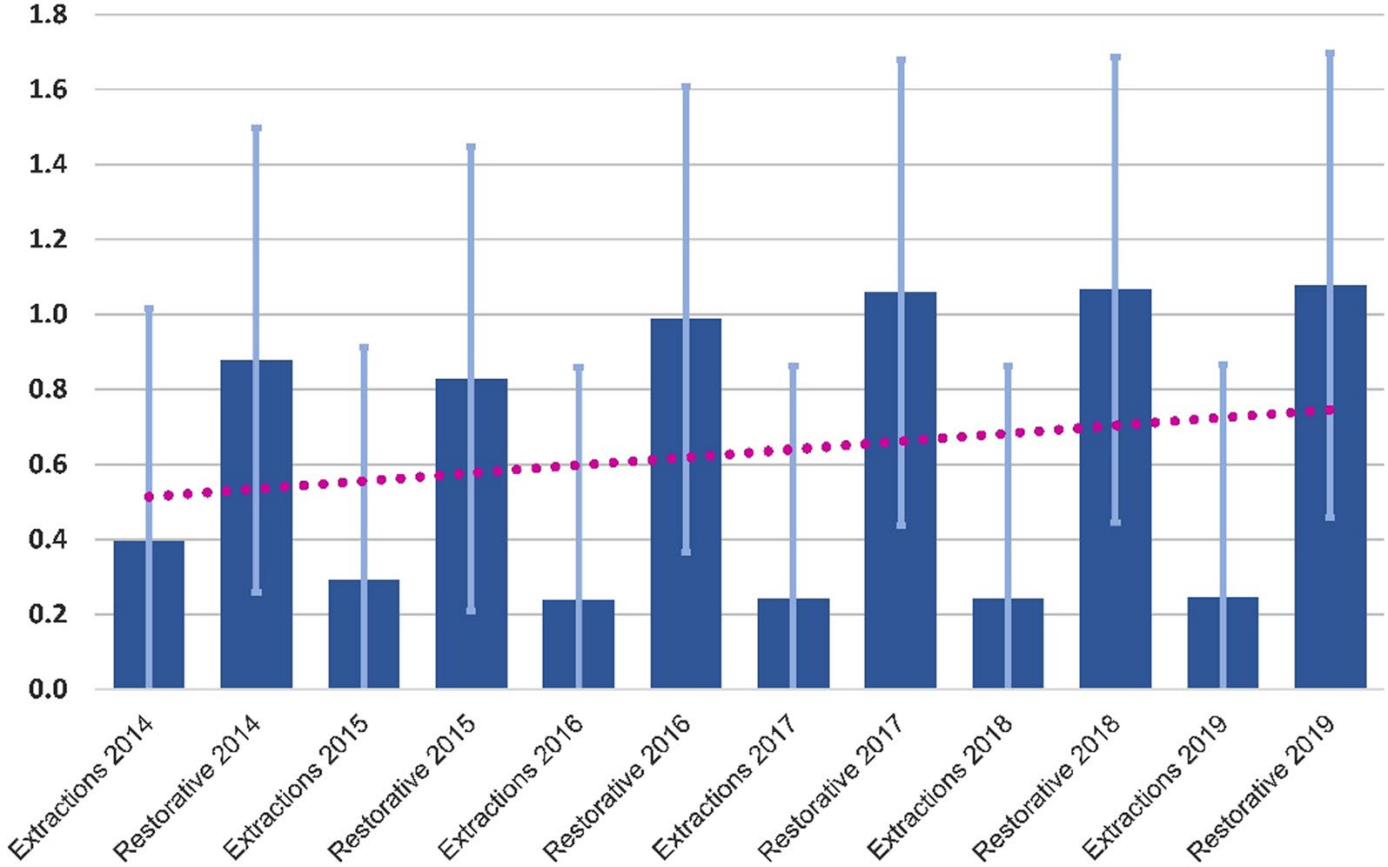
Dental treatment rates by years. Figure shows the constant increase in dental treatments from 2014 until 2019 (R^2^ = 0.0402)

Figure 1 shows that the number of dental treatments in 2014 and 2015 (when there was fluoridation and immediately after) did not change with age. However, between 2016 and 2019 the number of treatments increased with age.

**Fig. 2:**
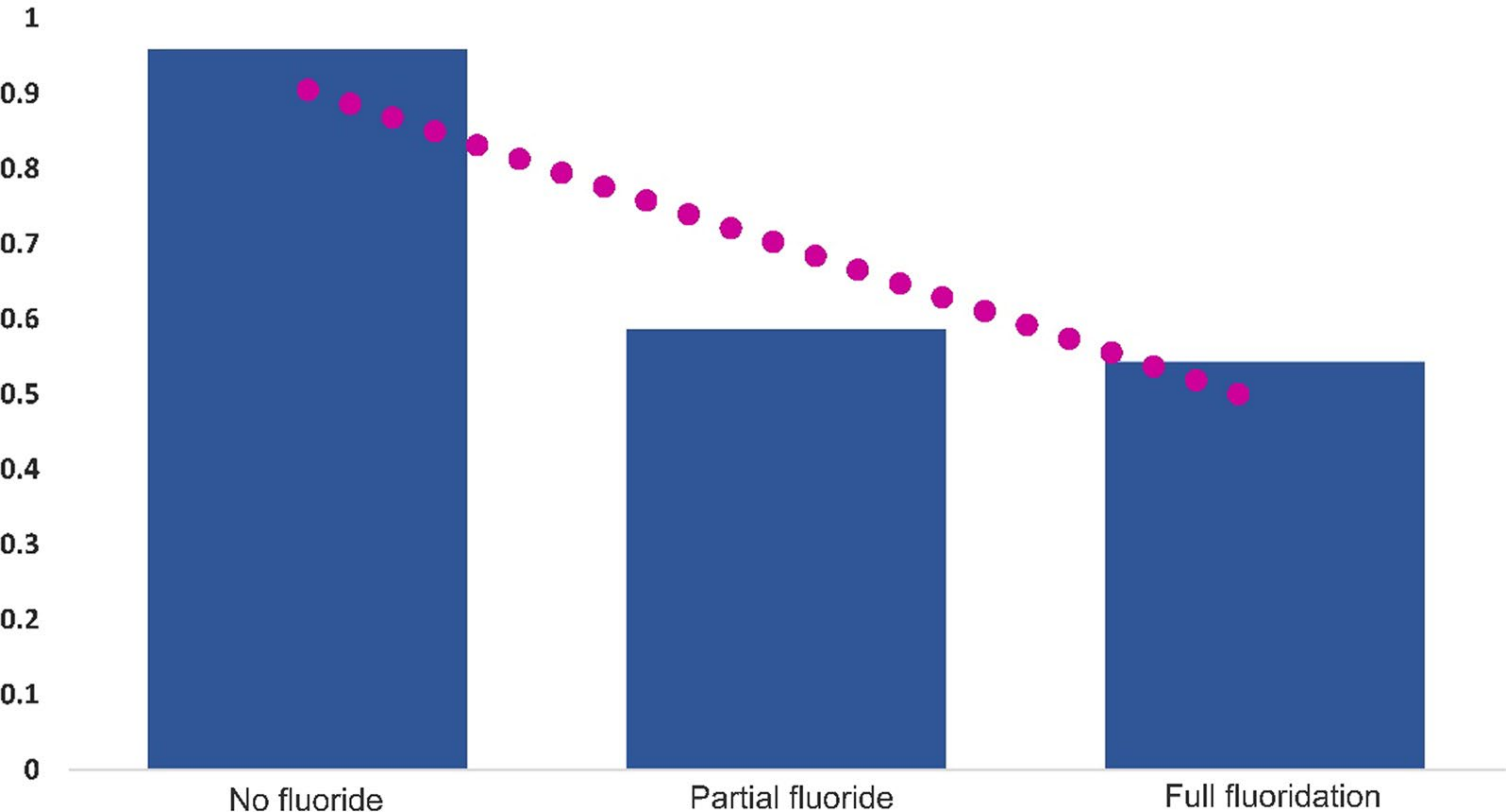
Dental treatment rates by fluoride levels in 2014. Figure shows low-rate treatments in 2014 in full fluoridated areas versus no fluoridated areas (R^2^ = 0.838)

Level of treatment was strongly related to, and explained by, CWF (R^2^ = 0.838). The rate of treatment was about double in the absence of CWF (Fig. 2).

### Analytic data

**Fig. 3:**
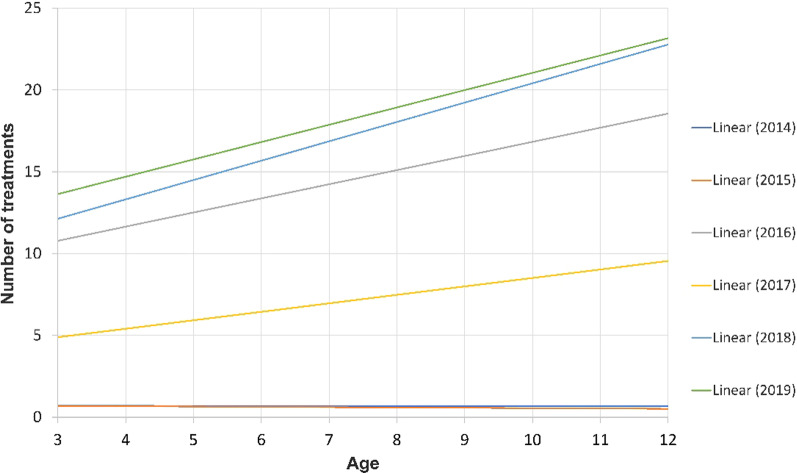
Mean cumulative number of dental treatments over time. Figure shows that when fluoridation was ceased, cumulative treatments rate started to rise

Linear regression was applied, and the number of treatments were the dependent variables, while age, CWF and SEP served as the independent variables. For the year 2014, when optimal fluoridation was present, the only significant variable related to extractions was CWF (β = − 0.096, *p* = 0.021). There were similar results restorative variables, i.e., only CWF was significant {F (1,458) = 4.336, *p* = 0.019} and for total treatments delivered (β = − 0.097, *p* = 0.019). The other independent variables did not reach statistical significance. For the years 2015–2019, when CWF ceased, the linear regression model showed opposite trends, with significant associations of age (β = − 0.389, *p* < 0.001) and SEP (β = 0.086, *p* = 0.019) (Fig. 3).

## Discussion

Over the years, there has been much interest in the issue of optimizing fluoride levels in drinking water with opposition from some human rights and ecological organizations [27]. Their expressed concerns, have often been based upon research that was of poor quality, misinterpreted, or contradicted by better studies, with more reliable findings. Public health officials, dental and medical practitioners and scientists have voiced steadfast support of CWF [28].

As mentioned above, water fluoridation began in Israel in 1981 [29–31]. At that time, about 90% of children suffered from tooth decay, and "rampant" caries was common among preschool children. There were very limited public dental health services, no free dental care available, and fluoride-containing toothpastes could not be obtained. Tooth decay rates increased from the 1950s, and a slight decline was noted in the 1970s when fluoride toothpastes came onto the market. With support from the WHO and a professional lobby, fluoridation of the water in Israel began in 1981. From 2001 the legislature demanded that communities of over 5000 people have CWF. Unfortunately, some communities did not receive optimally fluoridated water due to logistical factors. The decision to stop CWF in 2014 [32] was surprising in light of the consistent support shown.

A recent survey [27] on water fluoridation and dental health in Israel conducted by researchers from the Department of Community Dentistry, Hebrew University-Hadassah School of Dental Medicine, in 2011–2012 examined over 2000 twelve-year-old children and found a statistically significant difference in the rates of caries prevalence between fluoridated and non-fluoridated areas. A DMFT level of 0.98 was found in children living in fluoridated areas compared to 1.38 in those in non-fluoridated areas. The odds of being caries free were doubled in those living in fluoridated areas compared to non-fluoridated areas (OR = 2.09).

The 2010 dental reform to the National Health Insurance Law has increased access and utilization of dental care. The filled component of the DMFT index has increased while the decay component has decreased [22]. Still the overall prevalence rate of DMFT increases in the absence of water fluoridation and inequalities in oral health remains.

Other studies [33, 34] found that cessation of community water fluoridation appears to have negative effect on dental caries. McLaren and Singhal [33] published a systematic review in 2016 and concluded that “Overall, the published research points more to an increase in dental caries post-CWF cessation than otherwise”. Another study, Meyer et al. (2018) regarding consequences of community water fluoridation cessation in Alaska found that CWF cessation led to higher mean number of caries related procedures among 0–18 years old patients [34].

The present study was based upon data from 847,548 records of children aged 3–12 years visiting "Maccabi-Dent" clinics between 2014 (the last year of fluoridation) and 2019.

In 2014, 63.3% of the country was fully fluoridated, and the remaining areas were partially or completely unfluoridated.

When examining the data from 2014, in the localities with an optimal level of fluoridation had a lower number of treatments than the areas that were partially or completely unfluoridated. The treatment rate was found to be significantly lower for all treatment types, i.e. (restorative treatments or extractions).

This study included over 800,000 dental records, and the data supports the benefits of CWF in reducing caries. To the best of our knowledge, this is the largest database examined regarding fluoridation. We also confirmed the positive relationship between age and caries; with older children needing more treatments as shown in Fig. 3. We also found that the treatment rate correlated with fluoride levels. Our results clearly show the benefits of CWF in maintaining pediatric dental health. It seems that CWF was stopped for political reasons, and the lack of fluoride has led to an increase in dental problems which can cause systemic health issues.

### Limitation

The database we used did not provide the specific diagnoses, and only contained a record of the treatments performed. We assumed that the performance of treatments indicated the presence of disease, which is not always the case in fee for service payment systems such as in Maccabi-dent.

## Conclusion

This study found that water fluoridation at 0.7–1.2 ppm reduces the rates of treatment for caries, demonstrating the importance of CWF in oral health promotion. We call for immediate renewal of water fluoridation in Israel. An issue which is unequivocal, those who object to water fluoridation always have the alternative of using non fluoridated bottled water. Re-establishment of water fluoridation would need the support of the Ministry of Health which in turn would have to approach the Israeli Parliament for both final approval and funding. Since a high percentage of drinking water would be desalinated, fluoridation would have to be established also, in the desalination institutes.
